# The interface of condensates of the hnRNPA1 low-complexity domain promotes formation of amyloid fibrils

**DOI:** 10.1038/s41557-023-01289-9

**Published:** 2023-09-25

**Authors:** Miriam Linsenmeier, Lenka Faltova, Chiara Morelli, Umberto Capasso Palmiero, Charlotte Seiffert, Andreas M. Küffner, Dorothea Pinotsi, Jiangtao Zhou, Raffaele Mezzenga, Paolo Arosio

**Affiliations:** 1 Department of Chemistry and Applied Sciences, Institute for Chemical and Bioengineering, ETH Zurich, Zurich, Switzerland; 2 Scientific Center for Optical and Electron Microscopy, ETH Zurich, Zurich, Switzerland; 3 Department for Health Sciences and Technology, Institute of Food, Nutrition and Health, ETH Zurich, Zurich, Switzerland; 4 Bringing Materials to Life Initiative, ETH Zurich, Zurich, Switzerland

**Keywords:** Supramolecular assembly, Proteins

## Abstract

The maturation of liquid-like protein condensates into amyloid fibrils has been associated with several neurodegenerative diseases. However, the molecular mechanisms underlying this liquid-to-solid transition have remained largely unclear. Here we analyse the amyloid formation mediated by condensation of the low-complexity domain of hnRNPA1, a protein involved in amyotrophic lateral sclerosis. We show that phase separation and fibrillization are connected but distinct processes that are modulated by different regions of the protein sequence. By monitoring the spatial and temporal evolution of amyloid formation we demonstrate that the formation of fibrils does not occur homogeneously inside the droplets but is promoted at the interface of the condensates. We further show that coating the interface of the droplets with surfactant molecules inhibits fibril formation. Our results reveal that the interface of biomolecular condensates of hnRNPA1 promotes fibril formation, therefore suggesting interfaces as a potential novel therapeutic target against the formation of aberrant amyloids mediated by condensation.

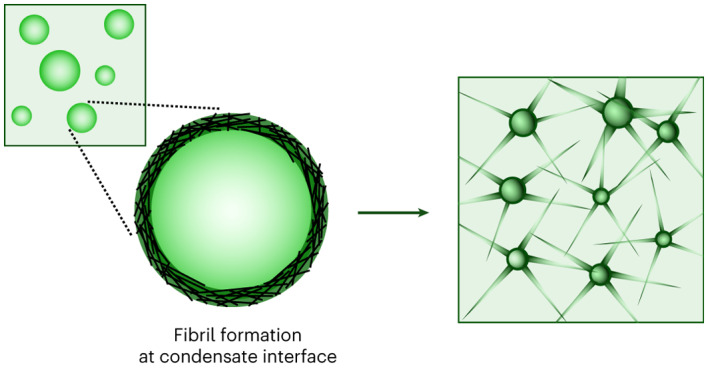

## Abstract (web summary)

Understanding of the molecular mechanisms underlying the maturation of protein condensates into amyloid fibrils associated with neurodegenerative diseases has so far remained elusive. Now it has been shown that in condensates formed by the low-complexity domain of the amyotrophic lateral sclerosis-associated protein hnRNPA1, fibril formation is promoted at the interface, which provides a potential therapeutic target for counteracting aberrant protein aggregation.

## Main

Recent findings indicate that cells can regulate functions in space and time via membraneless organelles composed of proteins and nucleic acids^1–3^. These viscoelastic condensates^4^ exhibit a variety of material properties, manifesting physical states that range from liquid-like^5–7^ to dynamically arrested^8,9^.

In some cases dynamic condensates exhibit a maturation process over time towards either arrested states^8,10,11^ or amyloid fibrils^12–15^. While for some condensates this ‘ageing’ can be functional^16^, in other cases liquid-to-solid transitions can lead to aberrant behaviour, as often observed with maturation into amyloids^17,18^.

Transitions from liquid-like condensates to amyloids have been observed for peptides^19,20^, α-synuclein^21^, tau^22,23^ and a variety of RNA-binding proteins (RBPs) associated with amyotrophic lateral sclerosis (ALS) and frontotemporal dementia^24,25^, including transactive response DNA binding protein 43 (TDP-43) (ref. ^26^), fused in sarcoma (FUS)^12^ and heterogeneous nuclear ribonucleoprotein A1 and A2 (hnRNPA1/2) (refs. ^13,27^).

Such RBPs have been found in insoluble cytoplasmic inclusions in neurons of post-mortem brain and spinal cord tissue of patients with ALS^24,28–30^ and have been shown to form amyloid fibrils with characteristic cross-β-sheet architecture^31^ in vitro^32–34^ and ex vivo^35^. These proteins have also been associated with the formation of several membraneless organelles including stress granules, Cajal bodies and paraspeckles exhibiting important functions in RNA metabolism^13,36,37^.

Recent effort has been focusing on unravelling the molecular modulators of the liquid–amyloid transitions of different RBPs. In vitro studies have shown that ALS-associated amino acid substitutions and post-translational modifications affect the liquid–amyloid transition of these RBPs. For instance, the balance of FUS phase separation and amyloid formation is modulated by phosphorylation and methylation^12,38,39^.

Together, these findings have supported the hypothesis that aberrant, misregulated liquid–amyloid transitions could play an important role in some amyloid-associated diseases^40^.

Despite the evidence that the formation of liquid-like condensates or the recruitment of aggregation-prone proteins therein could be an event promoting fibril formation^40,41^, the molecular mechanisms underlying such liquid–amyloid transitions are only starting to be understood. Unravelling these mechanisms has huge potential to identify effective therapeutic strategies to prevent the formation of aberrant fibrils^42^.

Here we analyse the liquid–amyloid transition of the intrinsically disordered region of hnRNPA1. hnRNPA1 consists of a globular domain containing two RNA-recognition motifs (RRM1 and RRM2) and a C-terminal prion-like, intrinsically disordered region, indicated as low-complexity domain (LCD). Both the liquid–liquid phase separation (LLPS) and the formation of amyloid fibrils of hnRNPA1 have been shown to be largely governed by the LCD^13,24^. Moreover, single point mutations, frameshifts and extensions of the LCD have been identified in patients with ALS and associated not only with mislocalization of the protein from the nucleus to the cytoplasm and subsequent accumulation therein, but also with altered LLPS, fibrillization and stress granule dynamics^24,43^.

The sequence of the LCD is very intriguing because it exhibits a peculiar ‘molecular grammar’ and contains regions that drive LLPS and others that promote amyloid formation. Specifically, LLPS is mediated by regularly spaced, aromatic amino acids that act as ‘sticker’ moieties^44,45^, while amyloid formation is mediated by zippers/low-complexity aromatic-rich kinked segments (LARKS)^46,47^ that promote β-sheet structures and reversible hydrogels at high protein concentrations in vitro^48^. This LCD is therefore an ideal model system to probe the correlation between LLPS and amyloid formation.

In this article, we demonstrate that LLPS and amyloid formation of the LCD of hnRNPA1 are correlated but distinct processes mediated by different regions of the protein and that amyloid formation is largely accelerated when initiated from condensates. By monitoring the temporal and spatial evolution of fibrillization with a variety of biophysical techniques, we show that the formation of hnRNPA1 amyloid fibrils from condensates is promoted at the interface of the droplets. We further show that targeting the interface is an efficient way to inhibit this liquid–amyloid transition.

## Results

### Distinct LCD regions independently mediate LLPS and fibrillization

**Fig. 1:**
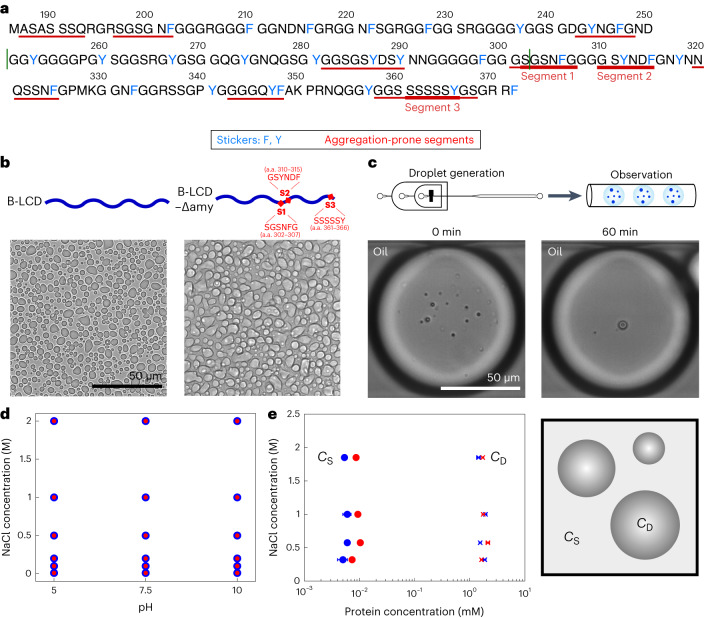
LLPS of B-LCD and variant B-LCD–Δamy. **a**, Sequence of the LCD of hnRNPA1-B (B-LCD). LCD of hnRNPA1-A (A-LCD) comprises amino acids (a.a.) 186–250 and 303–372 (borders indicated by green lines). Aromatic amino acids involved in LLPS are indicated in blue, while fibril-forming segments are underlined in red. **b**, Brightfield microscopy images of droplets formed by 30 µM B-LCD and 30 µM B-LCD–Δamy lacking three fibril-forming segments (ΔSGSNFG, ΔGSYNDF and ΔSSSSSY) in 50 mM Tris at pH 7.5 with 200 mM NaCl and 2 mM β-mercaptoethanol. Phase separation of B-LCD and B-LCD–Δamy was repeated independently at least three times with similar results. **c**, B-LCD droplets are liquid-like and merge in microfluidic water-in-oil droplet compartments until one single condensate is formed within 60 min. Microfluidic experiments have been repeated independently twice yielding similar results. **d**, Both 30 µM B-LCD (blue circles) and B-LCD–Δamy (red circles) undergo LLPS under a broad range of ionic strength and pH values. Circles indicate presence of LLPS. **e**, Protein concentrations inside (*C*_D_) and outside (*C*_S_) the droplets at different ionic strengths are similar for 30 µM B-LCD (blue) and B-LCD–Δamy (red). Error bars represent the standard deviation of the average protein concentration inside and outside the droplets, obtained by measuring three different droplets (for *C*_D_) using Raman spectroscopy and three independent samples (for *C*_S_) using centrifugation and UV absorbance (280 nm) at each NaCl concentration. When not visible, the error bars are smaller than the symbol. Source data

We investigate the C-terminal LCD of two splicing variants of hnRNPA1, isoforms hnRNPA1-A (A-LCD) and hnRNPA1-B (B-LCD), the latter containing 52 additional amino acids. The sequences of the proteins are shown in Fig. 1a, which highlights the aromatic residues that are responsible for LLPS^44^ and the regions that are prone to form amyloids (Methods and Supplementary Table 1).

In addition to the A- and B-LCD sequences, we generated a B-LCD variant that lacks three regions promoting amyloid formation: segment 1 (S1, ΔSGSNFG), which rapidly forms amyloid fibrils on its own^49^; segment 2 (S2, ΔGSYNDF) whose deletion in the LCD of isoform A effectively inhibits amyloid formation^13,50^; and segment 3 (S3, ΔSSSSSY), which exhibits the lowest interaction free energy according to ZipperDB (Supplementary Table 1). Segments S1 and S2 (Fig. 1a) overlap with the fibril core previously identified by cryo-electron microscopy^33^, and segments S1 and S3 have been predicted to exhibit LARKS-like properties (LARKSdb^46^). In the following we indicate this variant depleted of three fibril-forming segments S1–S3 as B-LCD–Δamy.

All variants exhibited phase separation over a broad range of buffer conditions, forming condensates immediately upon mixing the stock solution at higher urea concentration with a buffer containing 50 mM Tris at pH 7.5, 200 mM NaCl and 2 mM β-mercaptoethanol (Fig. 1b and Supplementary Fig. 1). We encapsulated the protein solution in water-in-oil microfluidic droplet compartments, which allows for accurate monitoring of coarsening events^51,52^. LCD droplets underwent coalescence until one single condensate per water-in-oil compartment was observed (Fig. 1c), therefore confirming the liquid-like nature of the condensates.

Importantly, the phase separation of all variants showed the same dependence on pH value and ionic strength (Fig. 1d and Supplementary Fig. 2). Additionally, we measured the concentration of the protein outside (*C*_S_) and inside (*C*_D_) the droplets at different ionic strengths. *C*_S_ was measured by ultraviolet (UV) absorbance after separating the droplet phase by centrifugation. *C*_D_ was measured by Raman spectroscopy, using a characteristic phenylalanine peak as standard^53^ (Methods and Supplementary Fig. 3). We observed very similar values for B-LCD and B-LCD–Δamy (Fig. 1e), confirming that the phase separation of the two constructs is nearly identical.

**Fig. 2:**
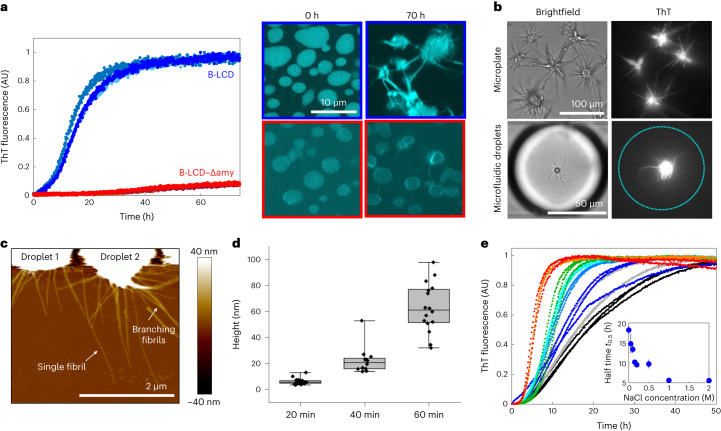
Liquid–amyloid transition of B-LCD is governed by fibril-forming segments. **a**, Fluorescence ThT signal over time for B-LCD (blue) and B-LCD–Δamy (red). Re-scan confocal microscopy images at time zero and after 70 h of incubation for the two constructs. Each curve represents the normalized ThT fluorescence of one independent sample. **b**, ThT-stainable star-shaped aggregates formed by B-LCD in microplates and in microfluidic water-in-oil droplets imaged in brightfield and by widefield fluorescence microscopy after 6 days of incubation. Starburst formation of B-LCD has been repeated at least five times in microplates and twice in a microfluidic set-up with similar results. **c**, AFM of star-shaped aggregates formed by B-LCD after 20 min. **d**, Increase of fibril height over 60 min after formation, extracted from AFM images. Box plots include the median fibril height, the interquartile range and the upper and lower quartile whiskers. For each timepoint, the height of 11–28 fibrils of 3–4 droplets was extracted. **e**, Acceleration of amyloid formation in 30 µM B-LCD sample with increasing NaCl (0 mM NaCl, black; 50 mM NaCl, grey; 100 mM NaCl, blue; 150 mM NaCl, light blue; 200 mM NaCl, cyan; 500 mM NaCl, green; 1 M NaCl, orange; 2 M NaCl, red). Inset: average half time *t*_0.5_ values as a function of NaCl concentration. Error bars represent the standard deviation of three independent samples. Source data

After characterizing the LLPS of the hnRNPA1 LCD variants at time zero, we monitored the formation of fibrils over time by a variety of biophysical techniques, including Thioflavin T (ThT) staining, optical microscopy, re-scan confocal microscopy, atomic force microscopy (AFM), Raman spectroscopy and transmission electron microscopy (TEM) (Fig. 2 and Supplementary Figs. 4 and 5). For both the A-LCD and B-LCD we observed an increase of the fluorescence ThT signal over time that follows the characteristic sigmoidal profile observed during amyloid formation (Fig. 2a and Supplementary Fig. 6), reflecting an increase in β-sheet-rich structures over time. By contrast, the increase in ThT signal over time for B-LCD–Δamy was negligible (Fig. 2a).

These results were consistent with re-scan confocal microscopy images acquired at the end of the incubation (∼70 h), which show the presence of ‘starburst structures’^12^ in A-LCD and B-LCD samples, and considerably fewer fibrils for B-LCD–Δamy (Fig. 2a and Supplementary Fig. 6). ‘Starburst structures’ formed by A-LCD and B-LCD in bulk assays and microfluidic compartments (Fig. 2b) are stainable by ThT and have been probed also by AFM (Fig. 2c) and TEM (Supplementary Fig. 5) analysis.

In microfluidic water-in-oil compartments, ‘starburst structures’ were observed already after 3 days of incubation (Fig. 2b), confirming not only that the time scale of liquid–amyloid transition is comparable with bulk experiments, but also that interfaces of the test tube do not play a major role in the liquid-to-solid transition.

Further analysis of AFM images revealed that after 20 min the fibril height is approximately 6 nm, corresponding to the typical diameter of an individual amyloid fibril (Fig. 2d). Over time, fibrils grew into thicker aggregates (Fig. 2d).

We also monitored the changes in the secondary structure of the protein over time by Raman spectroscopy. For B-LCD, spectra acquired at time 0 (condensates) and after several days (‘starburst structures’) showed a transition from a random coil state to β-sheet-rich structures^54,55^, while negligible changes were observed for B-LCD–Δamy (Supplementary Fig. 4).

Overall, the comparison between the LLPS behaviour (Fig. 1) and the liquid–amyloid transition (Fig. 2) of B-LCD and B-LCD–Δamy indicates that the two processes are mediated by different regions of the LCD: the deletion of segments that strongly drive the formation of amyloid fibrils does not impact phase separation.

This result is further supported by aggregation profiles measured at increasing concentrations of salt. While the LLPS is largely independent of the ionic strength of the buffer (Fig. 1e), amyloid formation is accelerated by increasing salt concentrations (Fig. 2e), indicating that the interactions responsible for liquid–amyloid transition and LLPS are different and can be independently modulated. The increase in ionic strength probably neutralizes an electrostatic repulsive interaction mediated by the residue D314 (D262 in the A-LCD) in segment S2, therefore accelerating fibril formation. This mechanism is consistent with the mutation D314V, which accelerates amyloid formation by substitution of this charged residue^24^. Re-scan confocal microscopy revealed that the morphology of B-LCD droplets and star-shaped aggregates is independent of the NaCl concentration (Supplementary Fig. 7).

### Amyloid formation is promoted at the condensate interface

We next investigated how condensate formation promotes the formation of amyloid fibrils. Since the kinetics of the liquid–amyloid transition of B-LCD was slower compared with A-LCD (Supplementary Fig. 6), in the following we decided to focus on B-LCD to better capture the transition process.

A first possible effect of condensation is the local increase of the protein concentration, which, from a kinetic angle, accelerates all microscopic nucleation and elongation reactions responsible for the formation of amyloids^56^.

**Fig. 3:**
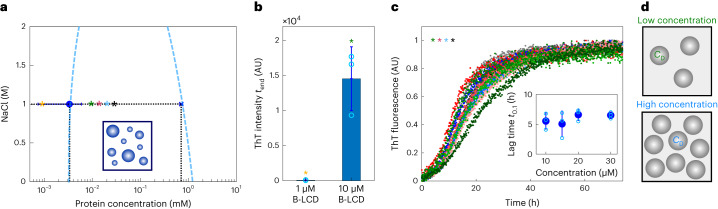
Condensation of B-LCD accelerates amyloid formation. **a**, Protein concentration inside (*C*_D_, blue cross) and outside (*C*_S_, blue circle) the condensates, measured as in Fig. 1. Asterisks indicate initial protein concentration in kinetic experiments in **b** and **c**: 1 µM (yellow), 10 µM (green), 15 µM (red), 20 µM (blue) and 30 µM (black). Error bars represent the standard deviation of the average protein concentration inside and outside the droplets, obtained by measuring three different droplets (for *C*_D_) and three independent samples (for *C*_S_) at each *C*_0_. The blue dashed line represents a guide to the eyes and indicates approximated upper and lower phase boundaries considering the total concentration of salt as a proxy for the intermolecular interaction parameter. **b**, Average ThT fluorescence intensity value of three replicates of 1 µM and 10 µM B-LCD solutions after 7 days incubation respectively below and above the critical concentration of LLPS (*C*_c_ = 2.5 µM). Error bars represent the standard deviation of the three replicates. **c**, Normalized ThT kinetic profiles corresponding to the initial protein concentrations *C*_0_ in **a**. The inset shows the average lag times *t*_0.1_ at various initial protein concentrations *C*_0_. Error bars represent standard deviation of three independent samples. **d**, Droplets formed at low and high initial B-LCD concentration exhibit similar internal protein concentration (*C*_D_) but increase in number when the initial protein concentration is increased. Source data

We incubated B-LCD samples at concentrations below the LLPS critical concentration (~2.5 µM, as shown in Fig. 3a), and we did not observe formation of amyloids over 7 days of incubation (Fig. 3b), indicating that for our system the formation of condensates is required to promote fibril formation or at least strongly accelerates the process. The absence of fibrils in samples containing B-LCD concentrations below the subcritical concentrations for LLPS was confirmed by TEM analysis (Supplementary Fig. 8).

By contrast, samples that contained B-LCD at concentrations above the critical LLPS concentration showed fibril formation within 2–3 days (Fig. 3a–c). Normalized ThT profiles collected at different initial protein concentrations were very similar (Fig. 3c). In contrast to the formation of amyloids from monomeric protein solutions, these data show that the lag phase is independent of the initial protein concentration when amyloid formation is initiated from a condensate state. This result can be explained by the fact that the protein concentration inside the droplets *C*_D_ is constant and independent of the initial protein concentration (*C*_0_) (Fig. 3a). Each individual droplet behaves as an individual microreactor at constant concentration *C*_D_. Increasing the initial protein concentration leads to an increase in the number of droplets (Supplementary Fig. 9) and in the total mass of fibrils formed, as shown by raw ThT profiles and the increase in the final ThT intensity values with increasing protein concentrations (Supplementary Fig. 10). However, the initial total concentration does not change the protein concentration inside the droplets (Fig. 3a), and therefore does not affect the lag phase (Fig. 3c). In the investigated concentration range, we did not observe any substantial change of the size distribution of droplets at different initial protein concentrations *C*_0_ (Fig. 3d and Supplementary Fig. 9).

**Fig. 4:**
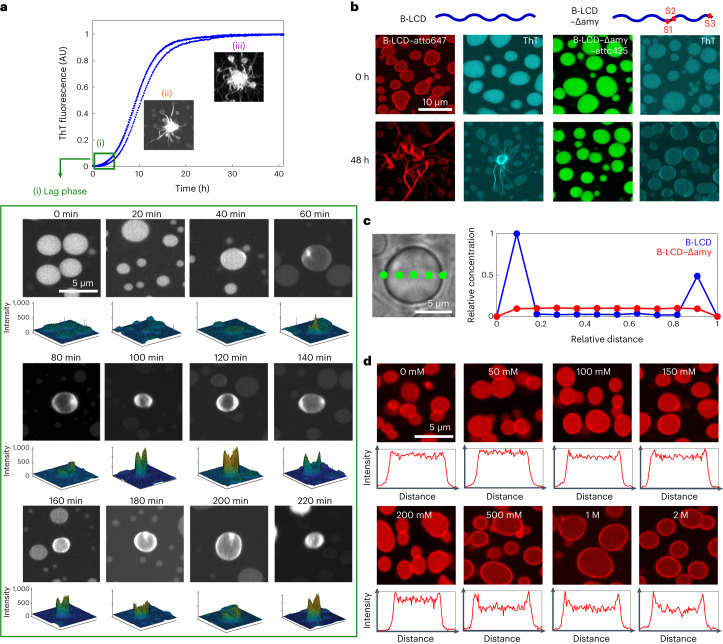
Formation of a ThT-positive protein-rich rim at the condensate interface precedes amyloid formation. **a**, Re-scan confocal fluorescence microscopy images collected along the ThT profile. During the initial lag phase (0–40 min) we observed the formation of the droplets and the uptake of ThT in their interior (i). Over time (60–220 min), a ThT-positive rim appeared on the droplet surface. Three-dimensional intensity profiles are depicted below each image, showing higher ThT intensity value at the droplet edge. During the growth phase, star-shaped aggregates were formed from the droplets (ii, iii). Micrographs in i–iii are representative images taken at each timepoint. **b**, Re-scan confocal fluorescence microscopy images of B-LCD labelled with atto647 at time 0 and after 48 h incubation. The rim at time 0 is not visible by ThT staining, while after 48 h the high ThT signal at the interface indicates the presence of β-sheet-rich structures. By contrast, droplets formed by B-LCD–Δamy did not exhibit accumulation of molecules or fibrils at the droplet interface. **c**, Representative protein concentration profile along the cross-section of one individual B-LCD droplet and one individual B-LCD–Δamy droplet as measured by Raman spectroscopy, confirming the increase in protein concentration at the droplet surface. **d**, Re-scan confocal fluorescence microscopy images of samples in **a** after 3 h of incubation. The rim is more evident for samples at higher NaCl concentrations, which exhibit faster kinetics of amyloid formation (Fig. 2e). The intensity profiles show the normalized droplet intensity as a function of the normalized droplet edge-to-edge distance for one representative droplet. Labelling and imaging of B-LCD and B-LCD–Δamy was performed three times yielding similar results. Source data

In addition to the temporal evolution of fibrillization, we next explored the spatial evolution of amyloid formation by performing re-scan confocal microscopy at different timepoints, while simultaneously monitoring the ThT profile (Fig. 4a). Initially, we observed recruitment of the ThT dye into the droplets, leading to low fluorescence intensity homogeneously distributed within the droplets due to high viscosity and increased concentration of the dye in the condensates (Fig. 4a). During the lag phase, microscopy images showed the formation of a ThT-positive rim on the surface of condensates, which grew into ThT-positive ‘starbursts’ during the growth phase (Fig. 4a).

To probe rim formation more in detail, we labelled both B-LCD (with atto647) and B-LCD–Δamy (with atto425) and performed re-scan confocal fluorescence microscopy. Images of B-LCD samples acquired a few minutes after preparation showed already the presence of a fluorescent rim along the droplet surface, which was not yet stainable by ThT (Fig. 4b). During incubation, the signal along this rim of individual droplets became ThT positive, indicating the formation of fibrillar structures (Fig. 4b). Importantly, all droplets which eventually formed ‘starburst structures’ exhibited a ThT-positive rim, indicating that fibril formation at the condensate rim is an initial event during the liquid–amyloid transition of hnRNPA1-B LCD condensates (Fig. 4b). Condensates formed by B-LCD–Δamy did not form ‘starbursts’. Only a slight increase of ThT fluorescence at the rim of almost all droplets was observed after 48 h (Fig. 4b). This increase of fluorescence probably occurs due to the remaining zippers/LARKS in the sequence of B-LCD–Δamy (Supplementary Table 1).

To ensure that the formation of this rim was not an artefact related to labelling the B-LCD with a fluorophore, we measured the concentration profile along an individual unlabelled B-LCD droplet by Raman spectroscopy 30 min after droplet formation. The results confirmed the increase of protein concentration at the surface of the droplet formed by B-LCD but not by B-LCD–Δamy (Fig. 4c), consistent with the results of re-scan confocal fluorescence microscopy (Fig. 4b).

The rate of the liquid–amyloid transition increased with increasing salt concentration (Fig. 2e). Consistently, re-scan confocal microscopy images acquired after 3 h incubation showed a more prominent rim for samples at higher salt concentrations (Fig. 4d).

In contrast to all the orthogonal data acquired with B-LCD, neither the increase in protein concentration at the droplet surface nor the formation of this ThT-positive rim could be observed in the B-LCD–Δamy samples. Only a very weak ThT signal along the droplet surface was visible after 48 h incubation (Fig. 4b), consistent with the flat ThT kinetic profile acquired in bulk (Fig. 2a).

Overall, these results demonstrate that the formation of amyloid fibrils in B-LCD droplets is promoted at the surface of the condensates. Moreover, these important findings indicate the surface as a potential target to arrest the transition from liquid droplets into amyloids.

**Fig. 5:**
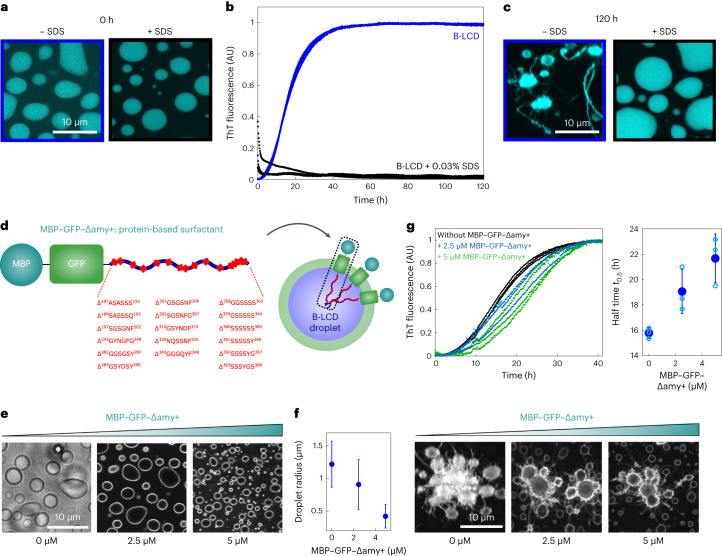
Targeting the surface of condensates inhibits amyloid formation. **a**–**c**, The addition of 0.03% SDS to a 30 µM B-LCD solution did not affect LLPS (**a**) but prevented the increase of ThT signal over time (**b**) as well as the formation of ThT-positive rims and star-shaped aggregates after 120 h, as shown by re-scan confocal microscopy images (**c**). Analysis of B-LCD droplets and starbursts in absence and presence of SDS was repeated three times yielding similar results. **d**, Design of a protein-based surfactant (MBP–GFP–Δamy+) consisting of soluble MBP, GFP and a B-LCD variant lacking all predicted steric zippers (Δamy+). **e**, Re-scan confocal fluorescence microscopy images showing accumulation of the protein-based surfactant molecules at the droplet surface. The fluorescent signal is originating from the GFP domain of the protein surfactant. Images were acquired at time 0. Experiments were independently repeated three times with similar results. **f**, Decrease of the average droplet size with increasing MBP–GFP–Δamy+ concentration. Error bars represent the standard deviation of sizes of 1,128 (0 µM MBP–GFP–Δamy+), 1,561 (2.5 µM MBP–GFP–Δamy+) and 1,020 (5 µM MBP–GFP–Δamy+) droplets from three independent samples. **g**, In addition to changing the size of the condensates, increasing concentrations of the protein-based surfactant delays amyloid formation inside condensates. Average half times *t*_0.5_ were extracted from the ThT profiles as a function of the MBP–GFP–Δamy+ protein surfactant. Black, 0 µM MBP–GFP–Δamy+; blue, 2.5 µM MBP–GFP–Δamy+; green, 5 µM MBP–GFP–Δamy+. Error bars represent the standard deviation of three independent samples. Re-scan confocal fluorescence microscopy images of samples after 40 h of incubation with increasing protein surfactant concentration. Source data

To confirm this hypothesis, as proof of concept, we aimed at delaying fibril formation by targeting the surface with two complementary strategies. In a first approach, we supplemented protein samples with 0.03% sodium dodecyl sulfate (SDS), one of the most common tensioactive agents. We monitored amyloid formation by ThT staining and fluorescence microscopy. The surfactant did not affect LLPS and the formation of liquid droplets, but drastically inhibited the increase of ThT signal and the formation of star-shaped aggregates over time (Fig. 5a–c).

In a second approach, inspired by the strategy described by Kelley et al.^57^, we generated a protein surfactant consisting of multiple domains: two soluble domains (maltose-binding protein (MBP) and green fluorescent protein (GFP)) coupled to a B-LCD variant that lacks many segments predicted to form amyloids (indicated in the following as Δamy+) (Fig. 5d and Supplementary Table 2). This protein is expected to accumulate at the interface, with the Δamy+ LCD facing the interior of the condensates and the soluble domains exposed to the solution (Fig. 5d). Re-scan confocal microscopy analysis confirmed preferential accumulation of the surfactant protein at the interface of the droplets (Fig. 5e) and the decrease of the average size of the droplets in the presence of increasing concentrations of the protein surfactant (Fig. 5f). Fibril formation inside condensates coated with this protein was delayed, as demonstrated by the increase of the half times *t*_0.5_ (Fig. 5g and Supplementary Fig. 11), confirming that interfaces are crucial for fibril formation from condensates of the LCD of hnRNPA1. The surfactant protein affected the liquid–amyloid transition of B-LCD condensates to a smaller extent than SDS, which possibly is partially present also in the interior of the condensate, potentially further contributing to stabilizing the condensate state. The concentration of protein in the dilute and dense phase was, however, similar in the absence and presence of the two surfactants (Supplementary Fig. 12) confirming that the inhibition/delay of amyloid formation is not due to a decrease of the protein concentration inside the condensates.

## Discussion

The LCD of hnRNPA1 exhibits a peculiar ‘molecular grammar’, comprising regions with sticker-spacer architecture that promote LLPS^44,45^ and aggregation-prone regions that induce fibril formation. Amyloid formation and phase separation appear to be two independent processes, since disease-causing mutants show phase separation behaviour similar to wild-type hnRNPA1 (refs. ^13,27^). Moreover, fibrillization is not required for phase separation^13^. We note that in general this behaviour can be different for other phase separation mechanisms, such as liquid–liquid crystalline phase separation^58^.

Here we analysed the C-terminal LCD of two splicing variants of hnRNPA1, isoforms hnRNPA1-A (A-LCD) and hnRNPA1-B (B-LCD). As control, we generated a sequence lacking three important amyloid-promoting segments. We observed that wild-type and modified LCD have essentially identical phase separation behaviour (Fig. 1). However, amyloid formation is drastically reduced for the LCD lacking three fibril-forming segments (Fig. 2). Therefore, our data support the notion that phase separation and fibrillization are connected but distinct processes that are modulated by different regions of the protein sequence. This is also consistent with our finding that the thermodynamics of LLPS is insensitive to changes in the ionic strength, while the kinetics of fibril formation is accelerated at increasing salt concentrations. These observations demonstrate that the two processes are governed by different interactions that can be independently modulated (Figs. 1e and 2e).

Even though LLPS and fibrillization are decoupled processes, LLPS strongly promotes the latter (Fig. 3). Indeed, at concentrations lower than the LLPS saturation concentration (2.5 µM), fibril formation was not observed within 7 days. In our system, condensation is therefore required for fibrillization or at least strongly accelerates fibril formation. A first intuitive explanation of this observation is the local increase of protein concentration of approximately 1,000-fold inside the condensates. If the critical concentration for amyloids is higher than the protein concentration in the dilute phase, amyloid formation can be promoted locally inside the condensates, where the concentration is in the millimolar range (Fig. 1e). If the critical concentration required for fibril formation, which in test tubes is typically in the nano- and micromolar range,^59^ is lower than the dilute phase, condensates are kinetically metastable with respect to amyloids and can accelerate amyloid formation by increasing nucleation and growth rates^60–62^.

Our analysis of the spatial evolution of fibrillization inside the condensates shows that fibril formation is promoted at the interface (Fig. 4). This indicates that the interplay between condensation and fibril formation is beyond a simple increase of local protein concentration.

Previous studies have shown that proteins can exhibit different conformations inside and outside the condensates. For instance, the protein tau adopts a more extended conformational ensemble within droplets that exposes an aggregation-prone region, therefore promoting amyloid formation^23^.

In addition to conformational changes inside and outside the condensate, Farag et al. have recently shown that LCD molecules of hnRNPA1 are organized into small-world topologies inside condensates^63^. Specifically, proteins are more expanded at the interface compared to both the interior of the condensate and the dilute phase^63^. Moreover, molecules prefer to be oriented perpendicularly to the condensate interface^63^.

These observations provide a plausible explanation of the promotion of amyloid formation at the interface of the condensates reported in this work. The orientation and conformation expansion of the molecules at the interface probably bring aggregation-prone regions of hnRNPA1 molecules in close proximity and locally promote β-sheet formation. Fibrils can grow from the condensates by recruiting soluble monomers either from the dilute phase or from dissolution of smaller droplets.

According to this picture, the promotion of amyloid formation at the interface of the condensates could share common aspects with surface-induced protein aggregation in homogeneous solutions. Indeed, interfaces are generally well known to promote heterogeneous primary nucleation events by inducing protein adsorption, local increase of protein concentration and possible conformational changes which trigger aggregation^64–69^.

Another possible explanation of our findings is provided by the analysis of Garaizar et al.^70^, which shows how core–shell multiphase condensates can spontaneously form during ageing of single-component condensates that are initially homogeneous and liquid-like. This transition arises due to the differences in physicochemical properties between monomers and oligomers or fibrils formed over time.

In addition to promoting conformational changes and orientation^63^, interfaces of condensates can preferentially partition components^71^ and accelerate or inhibit fibrillization of client molecules^72^. Thus, interfaces can represent a special location for biochemical activity and contribute to the complex interplay between condensation and fibrillization.

Local increase of protein concentration inside condensates increases polymerization and aggregation rate, as observed for instance with actin^73^. However, condensates are not simple liquids but viscoelastic materials^4^ often composed of multiple components. Heterotypic buffering^42^ can prevent amyloid formation of both scaffold^42^ and client proteins^74^ in the condensates despite the high local concentration. In another case, amyloid formation inside α-synuclein condensates was promoted at the centre of the droplets^75^. These examples demonstrate that interactions and conformations can differ at the interface with respect to the bulk of the condensate, in a system-specific manner, further modulating the interplay between condensation and amyloid formation.

Consistent with our findings that fibrillization of the hnRNPA1 LCD is promoted at the interface of condensates, we have demonstrated that the manipulation of the condensate interface affects the kinetics of fibril formation, resulting in a delay or complete arrest of amyloid formation (Fig. 5).

**Fig. 6:**
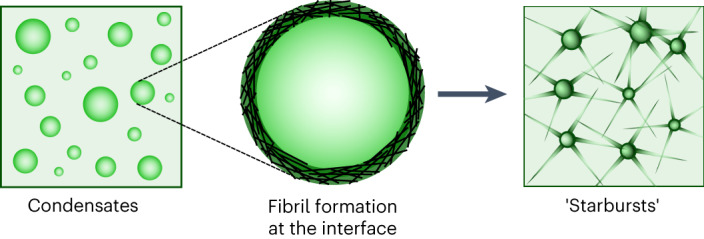
Promotion of amyloid formation at the interface of condensates. Schematic illustration of amyloid formation promoted at the interface of condensates of the LCD of hnRNPA1.

The results that we have presented here (Fig. 6) could have implications for the development of new therapeutic strategies aimed at arresting aberrant fibril formation mediated by condensation, and indicate condensate interfaces as a potential therapeutic target.

## Methods

### Protein expression and purification

hnRNPA1–A-LCD (15.2 kDa, sequence shown in Fig. 1a), hnRNPA1–B-LCD (19.8 kDa, sequence shown in Fig. 1a), hnRNPA1–B-LCD–Δamy (17.6 kDa, sequence shown in Fig. 1a) and MBP–GFP–Δamy+ were recombinantly expressed and purified in *Escherichia coli* BL21-Gold (DE3) cells. All DNA sequences were synthesized, codon optimized for expression in *E. coli*, and cloned into the pET-15b vector by GENEWIZ (Azenta). Plasmids containing the sequences for the different constructs, including N-terminal 6x-His tags, an isopropyl β-d-1-thiogalactopyranoside-inducible promoter as well as ampicillin resistance, were transformed via heat shock at 42 °C for 30 s. Cells were grown at 37 °C on Luria Broth (LB) agar plates containing 100 µg ml^−1^ ampicillin and further scaled up in rich media. For the MBP–GFP–Δamy+ surfactant, conventional LB medium was used. Protein expression was induced by addition of 0.5 mM isopropyl β-d-1-thiogalactopyranoside and incubation overnight at 37 °C. Following collection by centrifugation, cells were re-suspended in lysis buffer (8 M urea, 1 M NaCl, 50 mM Tris, pH 7.5 and 2 mM β-mercaptoethanol), lysed by sonication, and centrifuged at 18,300*g* for 20 min. The supernatant was transferred onto a Nickel NTA column, washed with wash buffer (1 M NaCl, 50 mM Tris, pH 7.5, 50 mM imidazole and 2 mM β-mercaptoethanol) and eluted by increasing the imidazole concentration in the buffer to 500 mM. The eluate was further polished by size exclusion chromatography on a Superdex 75 column (Cytiva) using a buffer containing 8 M (for A-LCD and B-LCD–Δamy) or 2 M urea (for B-LCD), 1 M NaCl, 50 mM Tris, pH 7.5, 10% glycerol and 2 mM β-mercaptoethanol. The eluted fractions were tested by SDS–polyacrylamide gel electrophoresis and Coomassie blue staining and pure fractions were pooled and immediately used for analysis. The purified protein was always used freshly. A representative chromatogram and gel are shown in Supplementary Fig. 13. Typically, B-LCD and B-LCD–Δamy proteins were concentrated to 300 µM and 600 µM stock solutions, respectively, using spin filters (Merck, Millipore; molecular weight cut-off 10 kDa).

The protein-based surfactant MBP–GFP–Δamy+ was expressed and purified in the same way but by using urea-free buffers containing a reduced NaCl concentration of 500 mM. Size exclusion chromatography was carried out on a Superdex 200 column (Cytiva).

For labelling experiments, B-LCD and B-LCD–Δamy were expressed and purified as described before, but the Tris-containing SEC buffer was replaced by phosphate-buffered saline supplied with 1 M NaCl and 2 M or 8 M urea. Following the instructions of the manufacturer, the purified protein samples were supplied with 2 mg ml^−1^ atto647 or atto425 dye dissolved in 100% dimethyl sulfoxide and incubated overnight at room temperature. Subsequently, the free dye was separated from the atto-conjugated proteins by size exclusion chromatography on a Superdex 75 column (Cytiva).

### Phase separation, fluorescence imaging and confocal microscopy

Phase separation of the B-LCD and the B-LCD–Δamy proteins was induced in 384-well plates (Matriplate, Brooks) by diluting stock solutions containing urea to the desired protein concentrations in 50 mM Tris, pH 7.5, 2 mM β-mercaptoethanol and different NaCl concentrations. Final urea concentrations were 200 mM or 400 mM.

Brightfield and widefield fluorescence microscopy were carried out on a Nikon Ti2 Eclipse inverted microscope, equipped with an LEDHub light source (Omicron) and an Andor Zyla camera, using a 60× oil objective (Nikon, numerical aperture 1.4). Image acquisition was controlled using the MicroManager software (version 2.0 gamma).

Confocal microscopy was performed with a Nikon NSTORM (Nikon) system equipped with a Rescan Confocal Microscope RCM1 (Confocal.nl). We used an sCMOS camera (Orca Flash 4.0 V2) and a Nikon SR Apochromat TIRF objective 100×/1.49 with oil immersion. Laser excitation wavelengths used were 488 nm, and 647 nm, to excite the ThT, atto488 and atto647 dyes, respectively. The set-up was fully controlled, and image acquisition was performed using the NIS Elements 5.20 Advanced research software (Nikon). The implemented re-scan unit provides an enhancement in resolution from 240 nm to 170 nm (refs. ^76,77^).

Droplet size distributions and numbers were extracted using an in-house written program in MATLAB (version R2020a).

### Amyloid formation kinetics

Amyloid formation was monitored by recording the fluorescence intensity of ThT over time. To this aim, samples were supplied with 20 µM ThT and measured in 384-well plates (Matriplate, Brooks) on a ClarioStar plus plate reader (BMG Labtech). To avoid evaporation, the plates were sealed with sticky aluminium foil (Corning). Measurements were taken every 10 min over several days, by applying excitation at 450 nm and measuring emission at 490 nm. Measurements were controlled using the Reader Control and MARS softwares (BMG Labtech).

### Raman spectroscopy

Raman spectra were acquired on a confocal inverted microscope (Nikon Ti-E) connected to a Raman system (Horiba, LabRAM HR Evolution UV-VIS-NIR). The sample was excited using a 532 nm laser (Nd:YAG, Cobolt Samba cw single frequency) at a power of 75 mW and the Raman signal was detected with a Synapse EM-CCD detector (Horiba). Spectra acquisition was controlled using the LabVIEW 2020 software (Labspec 6.5, Multiwell, EasyNav, National Instruments).

The protein concentration inside the droplets was measured as previously described^53^. In brief, a characteristic peak at 1,005 cm^−1^ corresponding to the symmetric breathing of the benzyl ring of phenylalanine was used as internal standard. A standard curve was generated by correlating the concentration of free phenylalanine to the height of the characteristic peak at 1,005 cm^−1^. The intensity of this peak corresponds to the number of phenylalanines in the sample, which can be directly related to the protein concentration by a standard curve (Supplementary Fig. 3). The method was validated by measuring the concentration of bovine serum albumin solutions at different known concentrations. Data analysis was performed using an in-house written code in Python.

Baseline correction and peak fitting of measured Raman spectra were performed using the Wavemetrics IgorPro 9 software. To extract secondary structure contributions in droplets and starburst structures of B-LCD and B-LCD–Δ-amy, peak positions were fixed at 1,610 cm^−1^ (phenylalanine and tyrosine), 1,640 cm^−1^ (α-helix), 1,665 cm^−1^ (random coil) and 1,675 cm^−1^ (β-sheet) as previously described^54,78,79^.

### Quantification of the protein concentration in the dilute phase

To quantify the protein concentration in the dilute phase, we first induced condensate formation as described in the previous paragraphs and incubated 40 µl of the sample for 30 min at room temperature. Then, samples were centrifuged at 18,400*g* on a benchtop centrifuge at 25 °C for 30 min. Five microlitres of the supernatant was removed, and the protein concentration was determined by nanodrop (Nanodrop Lite, Thermo Scientific). All measurements were performed with three independent samples.

Alternatively, the supernatant was analysed using size exclusion chromatography coupled with in-line fluorescence detection. Five microlitres of the supernatant was carefully removed and diluted eight times in buffer with 2 M urea to suppress phase separation. Samples were analysed on an Agilent 1200 Series HPLC equipped with Superdex 75 column (10/300, GE Healthcare) equilibrated in buffer containing 1 M NaCl, 2 M urea, 50 mM Tris pH 7.5, 2 mM β-mercaptoethanol and 10% glycerol. Thirty microlitres of sample was injected at a flow rate of 0.3 ml min^−1^. Chromatograms were recorded by monitoring protein intrinsic fluorescence on a 1260 Infinity II Variable Wavelength Detector exciting at 280 nm and measuring emission at 350 nm. All conditions were measured in duplicates. Concentration was estimated by calculating the area under the chromatogram peak after background subtraction using a Python script.

### TEM

Phase separation of B-LCD was induced as described above. Ten microlitres of the phase-separated sample was spotted on a glow-discharged grid (Lacey Carbon Support Film Grids, 300 mesh, Gold, Agar Scientific). The sample was dried at room temperature and stained with a 2% uranyl acetate solution for 1 min. After washing with distilled water, the grid was imaged using a TFS Morgagni 268 microscope (software iTEM 5.2 and MorgagniUI Version 3) and data were analysed using ImageJ. For the images probing absence of fibrils below the saturation concentration, samples were incubated for 7 days at room temperature before spotting 4 µl of the sample on the grid.

### Quantification of the dense phase volume fraction

The volume fraction of the dense phase was estimated from *z*-stacks of protein droplets acquired using confocal microscopy. Samples were prepared in 384-well plates with glass bottom (Matriplate, Brooks) using protein labelled with atto488 at a final concentration of 30 µM. A total of four *z*-stacks per condition were acquired using a Leica TCS SP8 confocal microscopy with a Hamamatsu Orca Flash 4.0 cMOS camera and an AOBS laser system (HyD Detector). Each *z*-stack covered a surface of 8,545.15 µm^2^, and the distance between images in the vertical direction was 0.1 µm. Image acquisition was controlled using the Leica LAS X SP8 software (version 1.0). Data were analysed with a customized Python script by counting the number of pixels with intensity above an arbitrarily defined threshold for each image. From the volume of a voxel (0.00324 µm^3^), the area of the well (10.9 mm^2^), the sample volume (20 µl), and assuming that protein droplets are homogeneously distributed on the well area, we estimated the total volume fraction of the dense phase from each *z*-stack.

### Phase separation and liquid–solid transition in microfluidic water-in-oil droplets

Fabrication of master wafers and polydimethylsiloxane devices were performed by standard soft lithography techniques as previously described^51^. The resulting devices exhibited a flow focusing architecture containing three inlets, two mixing junctions and one outlet. At the first junction, a homogeneous solution of B-LCD (120 µM stock) in 50 mM Tris, pH 8.5, 2 M urea and 2 mM β-mercaptoethanol was mixed with a buffer containing 50 mM Tris at pH 7.5 to induce phase separation. At the second junction, this mixture was encapsulated into water-in-oil droplets using an HFE-7500 oil (3 M) supplied with 0.5% Pico-surf surfactant (Sphere Fluidics). To observe the droplets over hours or days, the water-in-oil compartments containing the phase-separated droplets were transferred into glass capillaries and tightly sealed. Images were taken by brightfield or epi-fluorescence microscopy.

### AFM

AFM imaging was carried out by a Nanoscope Multimode 8 scanning probe microscope (Bruker). Fifty microlitres of a freshly prepared B-LCD droplet solution was deposited and then adsorbed onto freshly cleaved mica for 5 min before imaging at room temperature. The imaging was operated under hydrated condition in a liquid cell with a V-shaped silicon nitride cantilever (Bruker) with a nominal spring constant of 0.7 N m^−1^ and tip radius of 2 nm. The AFM probe was scanned at a scan frequency of 0.4 Hz on the sample continuously to screen the evolution of microdroplets. AFM images were flattened using the Nanoscope 8.1 software (Bruker), and no further image processing was applied.

### Zipper and LARKS prediction using ZipperDB and LARKSdb

Steric zipper regions were predicted using the data base ZipperDB^80^ available online at https://services.mbi.ucla.edu/zipperdb/.

LARKS were predicted using the LARKSdb^46^ available online at https://srv.mbi.ucla.edu/LARKSdb/index.py.

### Reporting summary

Further information on research design is available in the Nature Portfolio Reporting Summary linked to this article.

## Online content

Any methods, additional references, Nature Portfolio reporting summaries, source data, extended data, supplementary information, acknowledgements, peer review information; details of author contributions and competing interests; and statements of data and code availability are available at 10.1038/s41557-023-01289-9.

### Supplementary information

Supplementary Information Supplementary Figs. 1–13 and Tables 1 and 2.

Reporting Summary

Supplementary Table Source data for Supplementary Information.

### Source data

Source Data Fig. 1–5 Source Data Fig. 1 Protein concentration quantification inside and outside of droplets, Source Data Fig. 2 ThT aggregation kinetics and AFM fibril height, Source Data Fig. 3 Protein concentration quantification inside and ouside of droplets, and ThT aggregation kinetics, Source Data Fig. 4 ThT aggregation kinetics, Raman spectroscopy and fluorescence intensity droplet cross-sections and Source Data Fig. 5 ThT aggregation kinetics in presence and absence of surfactants, and droplet radii.

## Data Availability

All data relevant for this manuscript have been uploaded on figshare and are publicly available (10.6084/m9.figshare.23262005.v1 and 10.6084/m9.figshare.23262008.v1). Raw micrographs are available from the corresponding author upon reasonable request. ZipperDB (https://services.mbi.ucla.edu/zipperdb/) and LARKSdb (https://srv.mbi.ucla.edu/LARKSdb/index.py) databases used in this work to identify fibril-forming segments are publicly accessible. Source data are provided with this paper.

## Code availability

All codes used in this study are deposited on figshare (10.6084/m9.figshare.23605407.v1).
