# Supplementary material for: The interface of condensates of the hnRNPA1 low-complexity domain promotes formation of amyloid fibrils
Source: Nat Chem. 2023 Sep 25;15(10):1340–9. doi: 10.1038/s41557-023-01289-9 (PMC10533390; doi:10.1038/s41557-023-01289-9)
Supplement: Supplementary file 1 — Supplementary Figs. 1–13 and Tables 1 and 2. [file 41557_2023_1289_MOESM1_ESM.pdf]

nature chemistry

Article https://doi.org/10.1038/s41557-023-01289-9

The interface of condensates of the hnRNPA1 low-complexity domain promotes formation of amyloid fibrils

In the format provided by the authors and unedited

Table of Contents

1. Supplementary Figures

Supplementary Figure 1: LLPS of A-LCD

Supplementary Figure 2: Phase diagrams B-LCD and B-LCD-Δamy

Supplementary Figure 3: C_D via Raman spectroscopy

Supplementary Figure 4: Raman spectra B-LCD and B-LCD-Δamy

Supplementary Figure 5: TEM B-LCD starbursts

Supplementary Figure 6: Liquid-amyloid transition kinetics A-LCD and B-LCD

Supplementary Figure 7: B-LCD droplets and starbursts at increasing salt concentrations

Supplementary Figure 8: TEM of sub-critical solutions

Supplementary Figure 9: B-LCD LLPS dependent on protein concentrations

Supplementary Figure 10: B-LCD, raw Thioflavin T liquid-amyloid transition kinetics

Supplementary Figure 11: B-LCD liquid-amyloid transition in presence of surfactant

Supplementary Figure 12: Validation of C_S and ϕ_D

Supplementary Figure 13: Purification of A-LCD, B-LCD and B-LCD-Δamy

2. Supplementary Tables

Supplementary Table 1: Prediction steric zipper regions using ZipperDB

Supplementary Table 2: Sequences of B-LCD and Δamy+

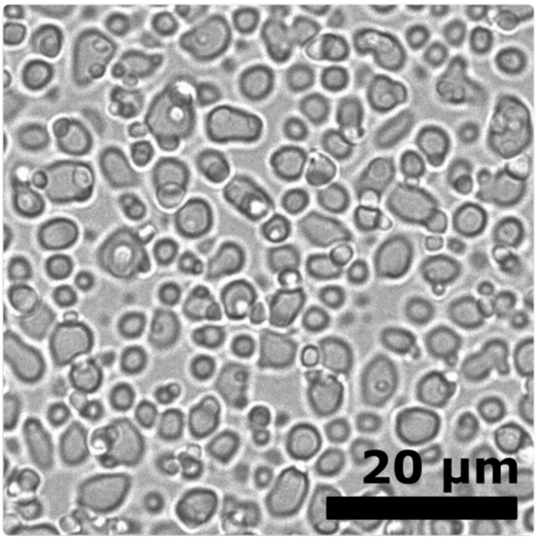

Supplementary Figure 1: The LCD of the isoform A of hnRNPA1 (A-LCD) undergoes liquid-liquid phase separation using 30 µM A-LCD in 50 mM Tris, pH 7.5, 200 mM NaCl, and 2 mM β-mercaptoethanol. Phase separation of A-LCD has been repeated at least three times with similar results.

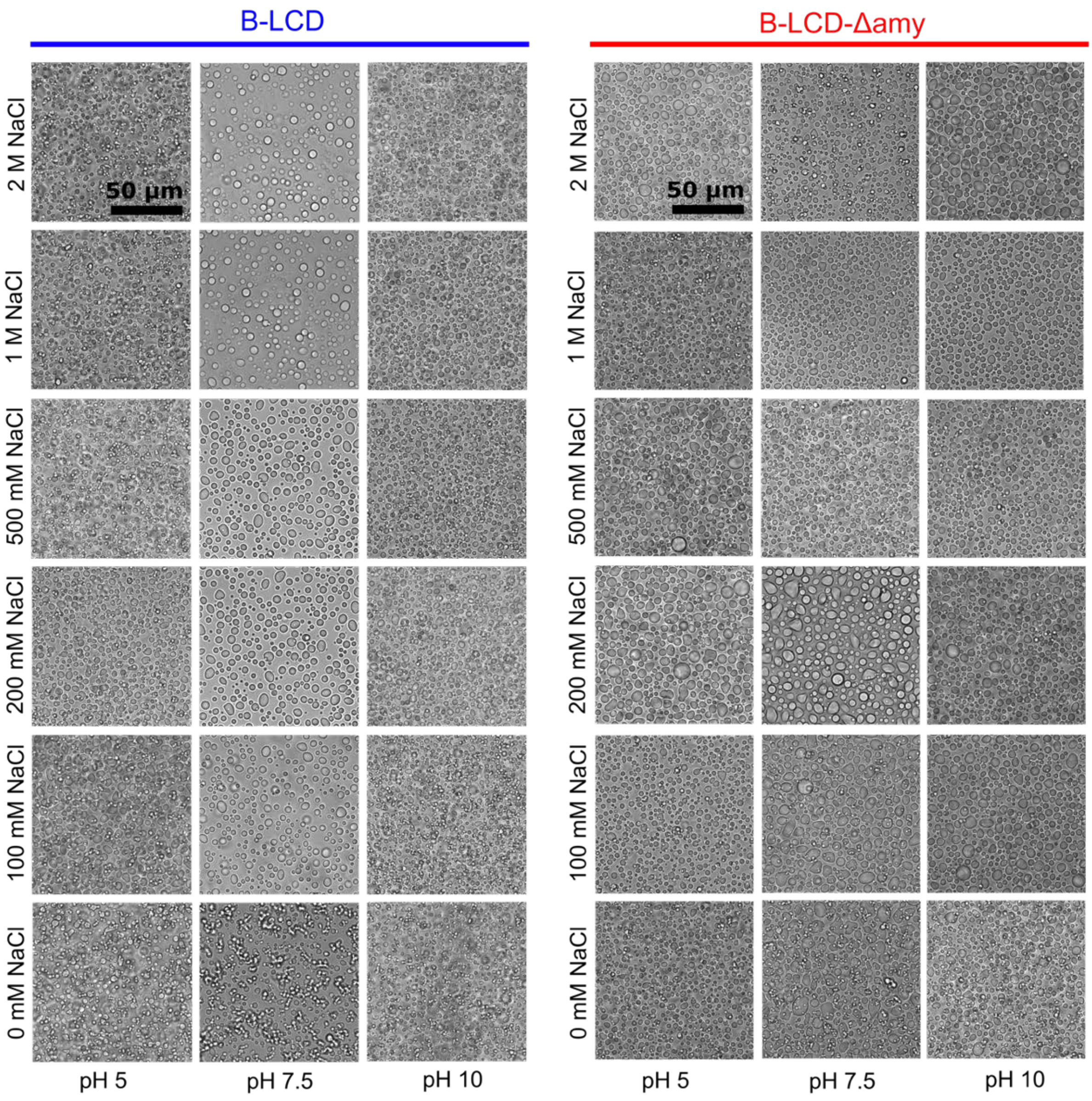

Supplementary Figure 2: Brightfield micrographs corresponding to the phase diagrams shown in Figure 1 in the main text. 30 µM B-LCD and B-LCD-Δamy in 50 mM Tris buffer at different NaCl concentrations and pH values. Even at extreme conditions both proteins form condensates that do not vary substantially in size and number, confirming that B-LCD and B-LCD-Δamy phase separation is mostly insensitive to pH and ionic strength. Both phase diagrams have been repeated independently three times with consistent results.

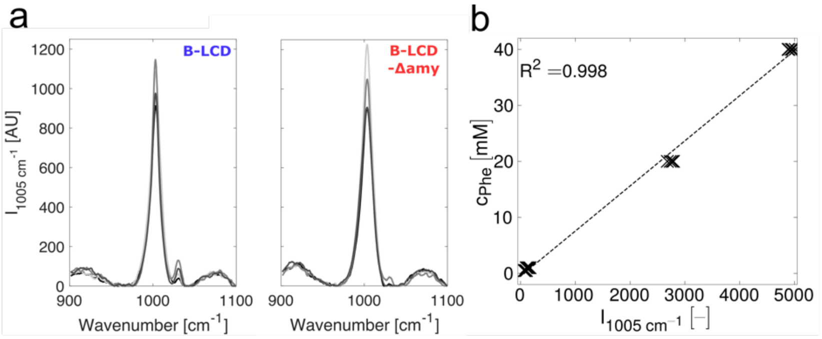

Supplementary Figure 3: Determination of the protein concentration inside condensates by Raman spectroscopy using a characteristic phenylalanine (Phe) peak at 1005 cm^{-1} as standard. **(a)** Representative Phe peaks of B-LCD (left) and B-LCD-Δamy droplets formed at 200 mM, 500 mM, 1 M and 2 M NaCl. The height of this Phe peak correlates linearly with the concentration of phenylalanine, i.e. with the protein concentration in the droplet. **(b)** Standard curves, obtained by measuring the peak height ($I_{1005\ cm^{-1}}$) of pure Phe solutions at known concentrations (1, 20 and 40 mM), measured at different Raman settings, confirming the linear relationship between signal and concentration. In this work, Raman spectra of the standard curves were recorded using the same settings as the Raman spectra of the droplets. Raman settings: 75 mW laser power, 532 nm, 2 x 120 s exposure, 1800 grating / cm.

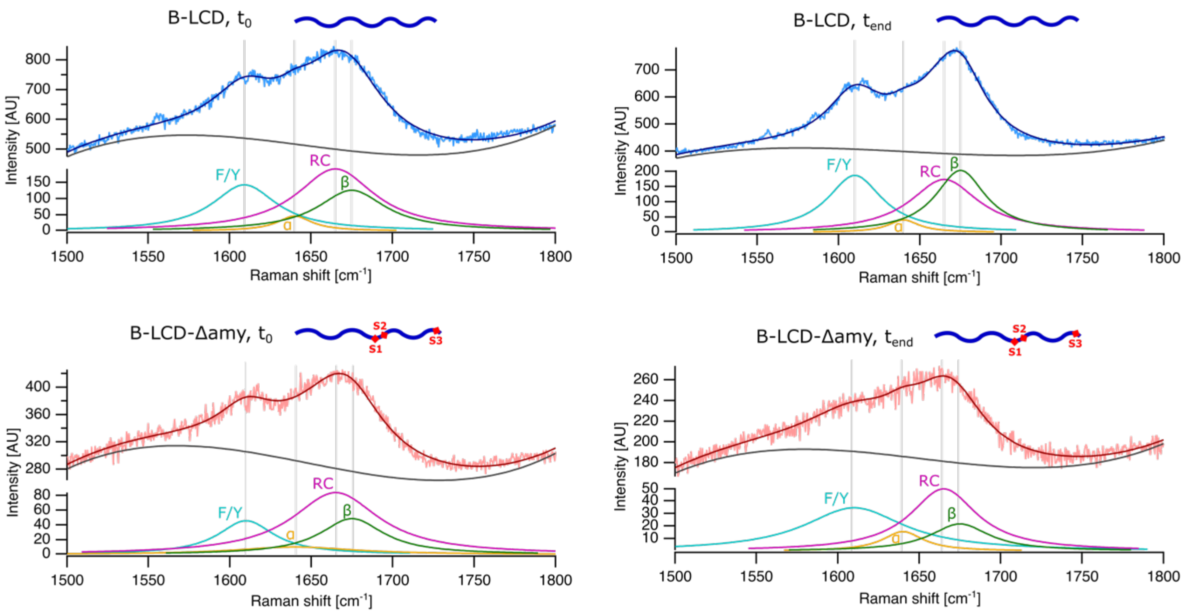

Peak position [cm^{-1}]	% Area B-LCD, t_0	% Area B-LCD, t_{end}	% Area Δamy, t_0	% Area Δamy, t_{end}
**1640**	6.3	5.3	10.5	12.1
**1665**	59.7	50.8	62.9	63.6
**1675**	34.0	43.9	26.7	24.3

Supplementary Figure 4: Raman spectra (Amide I regions) of B-LCD (upper panels) and B-LCD-Δamy (lower panels) condensates before and after incubation. t_0 corresponds to 30 min after droplet formation, while spectra at t_{end} were acquired 3 days after droplet formation (when the ThT fluorescence intensity reached plateau in the kinetic assays). Raw data is shown in blue (B-LCD) and red (B-LCD-Δamy). B-LCD underwent a shift of the Amide I peak from lower to higher wave numbers (1663 to 1673 cm^{-1}) which was absent in B-LCD-Δamy. While B-LCD samples at t_0 exhibit higher random coil contributions (RC, pink curve), B-LCD starburst structures at t_{end} exhibited higher β-sheet contributions (β, green curve). By contrast, B-LCD-Δamy condensates showed secondary structure contributions at t_0 comparable to B-LCD condensates that did not significantly change over time. F/Y – phenylalanine / tyrosine peak, 1610 cm^{-1}, blue; α – α-helix, 1640 cm^{-1}, yellow; RC – random coil, 1665 cm^{-1}, pink; β – β-sheet, 1675 cm^{-1}, green (Chatterjee S. et al., *Adv. Sci.* **9**, 2104247 (2022)).

We extracted the relative content of the three secondary structure elements probed in the Amide I region by calculating the respective peak areas. As seen in the table, the relative content of β-sheets (peak 1675 cm^{-1}) in condensates formed by B-LCD increases over time, while it remains essentially constant in case of the B-LCD-Δamy variant.

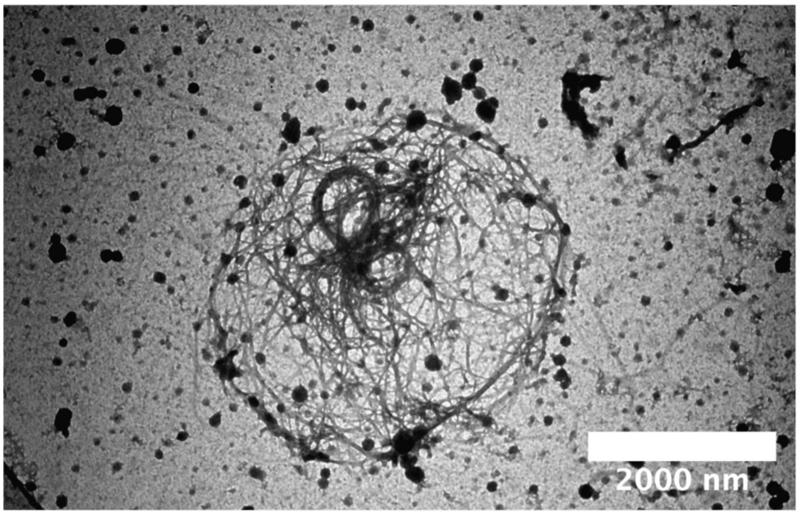

Supplementary Figure 5: Transmission electron micrograph of amyloid fibrils formed within B-LCD condensates. TEM was performed on three technical replicates, yielding similar results.

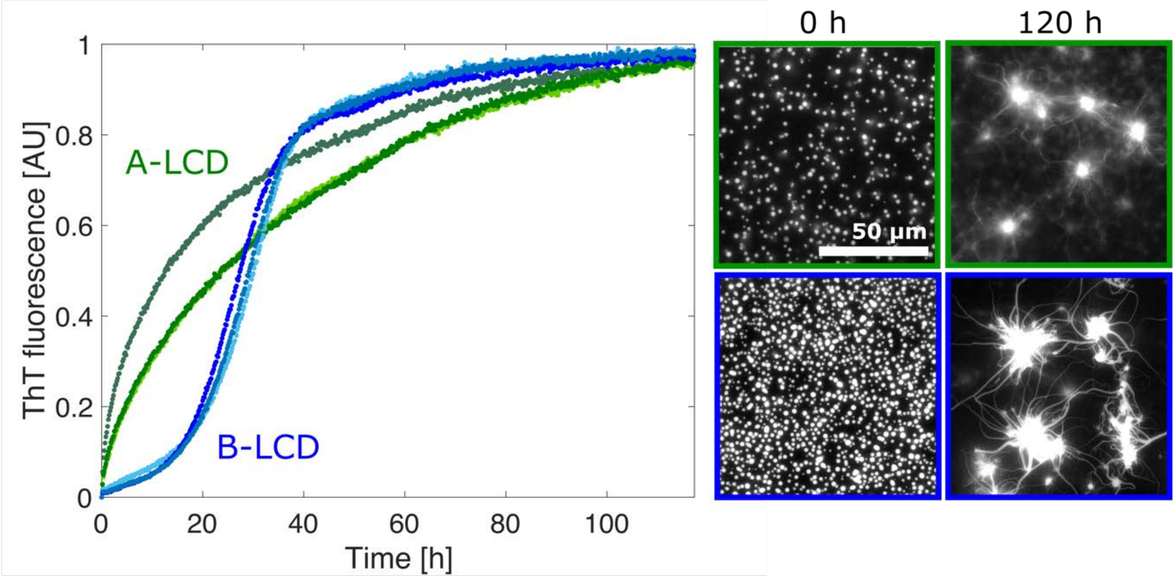

Supplementary Figure 6: Comparison of the liquid-amyloid transition of A-LCD and B-LCD. Both proteins undergo liquid-amyloid transition and form starburst structures over time. However, A-LCD aggregates faster than B-LCD. A-LCD and B-LCD liquid-amyloid transition experiments were repeated at least three times yielding comparable results.

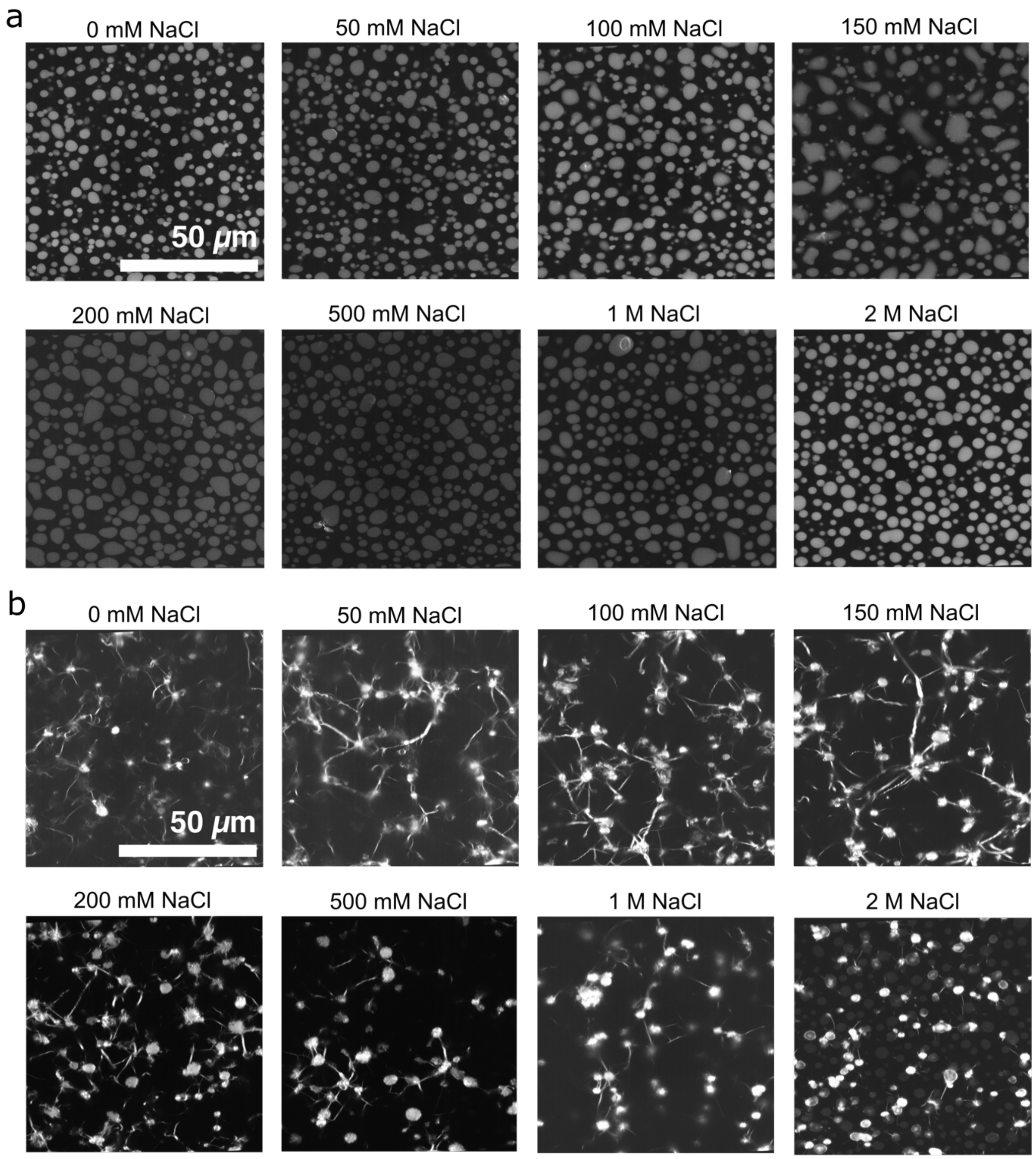

Supplementary Figure 7: Re-scan confocal microscopy of Thioflavin T-stained B-LCD droplets at time point 0 (a) and after 30 h incubation (b). Condensates were formed at a protein concentration of 30 µM, in 50 mM Tris, pH 7.5, 2 mM β-mercaptoethanol, 200 mM urea and various NaCl concentrations. Experiments in (a) and (b) were repeated at least three times yielding similar results.

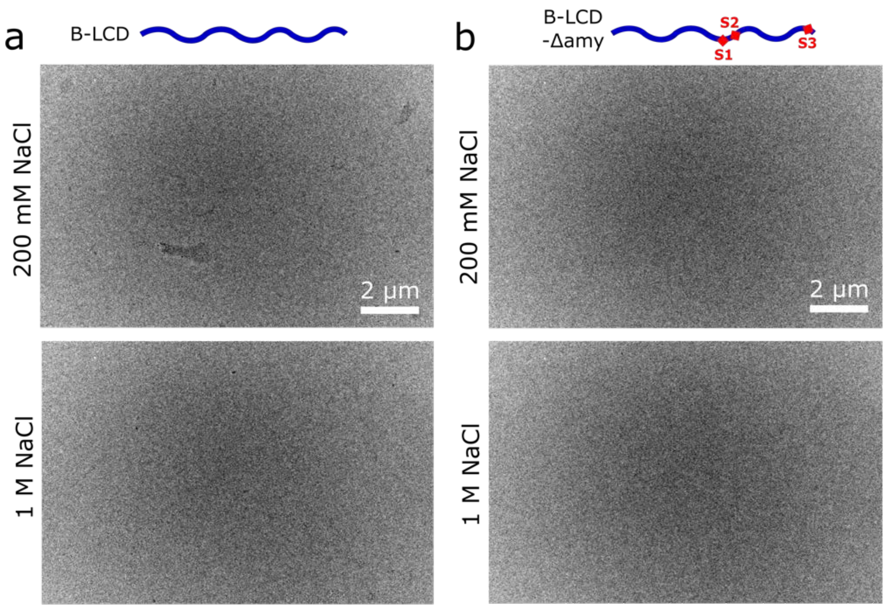

Supplementary Figure 8: TEM images after incubation for 4 days at sub-critical concentrations. (a) B-LCD at 200 mM and 1 M NaCl. (b) B-LCD-Δamy at 200 mM and 1 M NaCl. Neither the B-LCD (a) nor the B-LCD-Δamy (b) construct shows higher order structures. TEM was performed on three independent grids, yielding similar results.

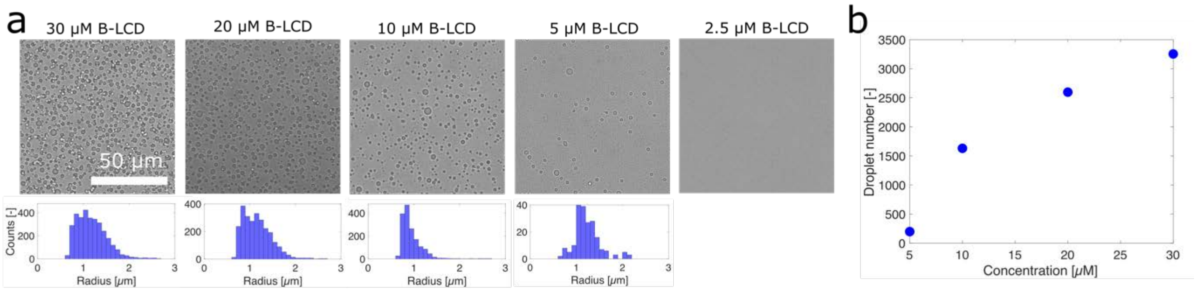

Supplementary Figure 9: (a) Images of B-LCD droplets formed at various initial protein concentrations acquired by brightfield optical microscopy and corresponding size distributions extracted from these images. **(b)** Number of droplets extracted from images in (a). Experiment was performed three times resulting in similar trends of droplet size distributions and droplet numbers.

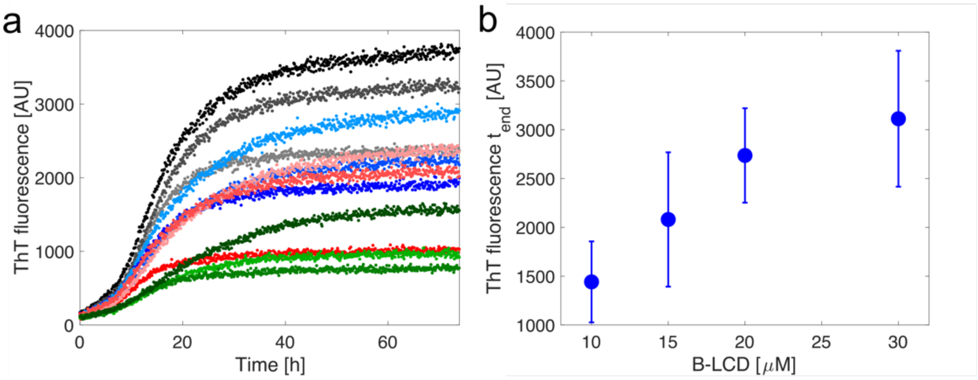

Supplementary Figure 10: (a) Raw Thioflavin T (ThT) kinetic data of B-LCD aggregation over time at different initial protein concentrations. 10 µM (green), 15 µM (red), 20 µM (blue), 30 µM (black). **(b)**. Average ThT intensity value after 70 h of incubation as a function of initial protein concentration. The error bar represents the standard deviation of three independent samples.

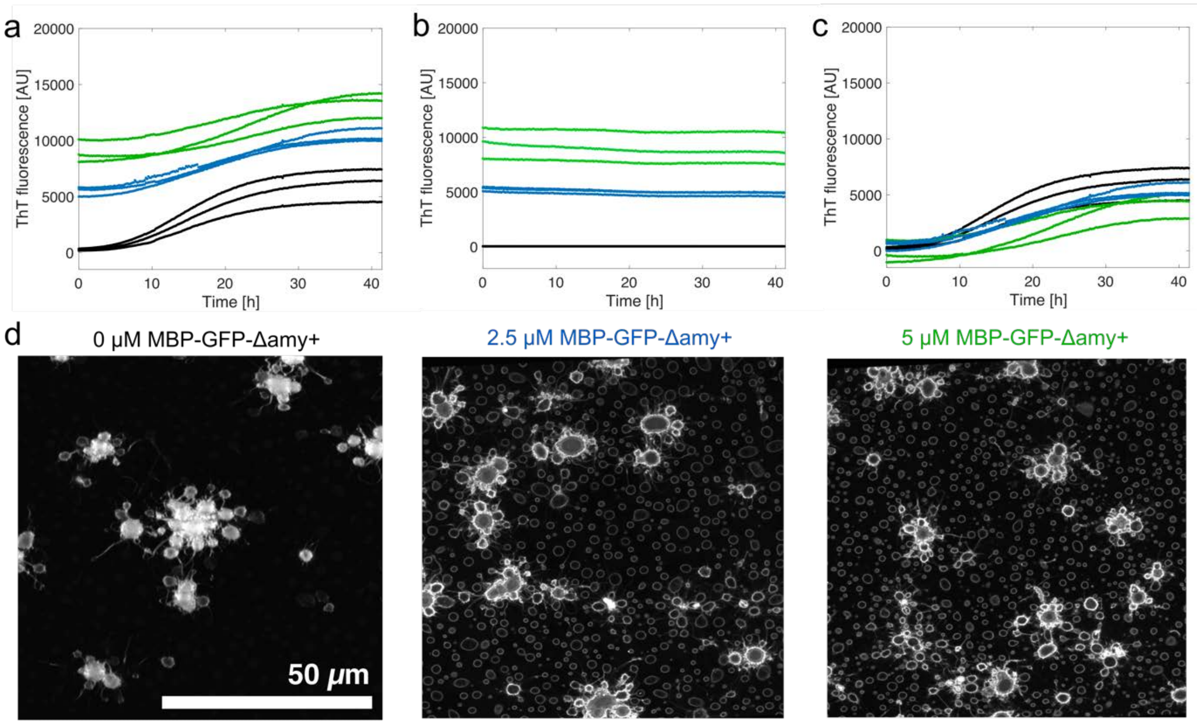

Supplementary Figure 11: (**a**) Time evolution of raw Thioflavin T fluorescence intensity value for 30 µM B-LCD sample in the absence (black), and presence of 2.5 (blue) and 5 µM (green) MBP-GFP-Δamy+ protein-based surfactant. Due to the similar emission wavelength of ThT and GFP, GFP fluorescence contributes to the recorded signal. **(b)** Recorded GFP signal of the same samples in (a) in the absence of ThT. GFP signal is constant over time, and increases with increasing amounts of surfactant. **(c)** Raw ThT signal from (A) subtracted by the average constant GFP signal in (b). **(d)** Re-scan confocal microscopy images of B-LCD aggregates in presence of increasing concentrations of MBP-GFP-Δamy+. Fluorescence signal contains the combined contributions from ThT and GFP. Amount and size of formed aggregates are similar in all samples. Experiments were independently repeated three times with similar results.

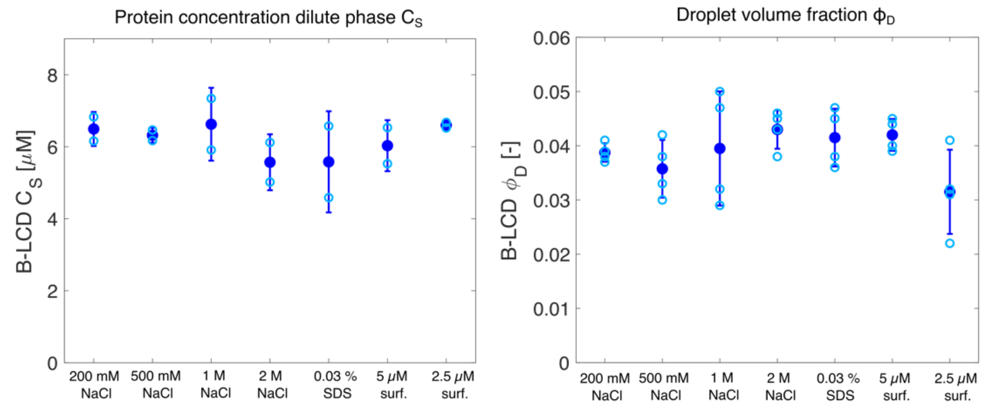

Supplementary Figure 12: Average concentration of protein in the dilute phase C_s (left) and average total volume fraction of the condensates ϕ_D (right) for 30 µM B-LCD samples. Based on a simple mass balance, the concentration of the protein in the dense phase is also similar under all conditions. Surf.: protein-based surfactant MBP-GFP-Δamy+ described in the main text. Error bars represent the standard deviation of 2-4 independent replicates.

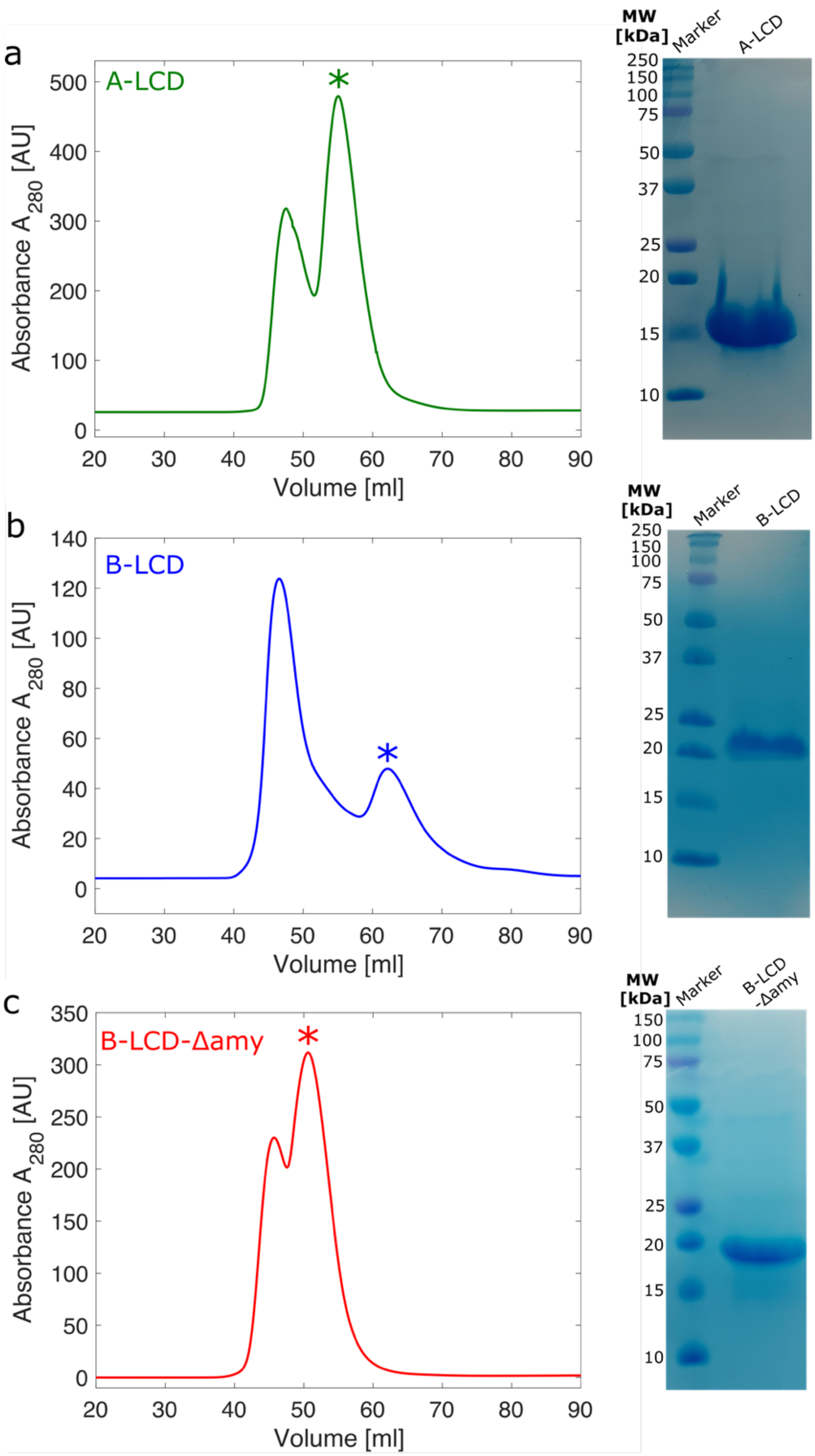

Supplementary Figure 13: Purification of A-LCD (a), B-LCD (b) and B-LCD-Δamy (c) by size exclusion chromatography. Asterisks in chromatograms show fractions considered for the experiments. Purity was verified by poly-acrylamide SDS-PAGE, followed by Coomassie staining. All constructs show the expected molecular weight and high purity. Purifications of A-LCD, B-LCD and B-LCD-Δamy were independently repeated at least ten times, yielding similar elution volumes and purity.

Supplementary Table 1: Steric zippers of the LCD of hnRNPA1-B, as predicted by ZipperDB. Highlighted in yellow are steric zipper sequences which have been deleted to obtain the less-aggregation prone variant B-LCD-Δamy. Steric zipper regions which overlap with LARKS are marked with an asterisk (*).

Position	Sequence	Rosetta Energy	Shape Compl.	Area of Interface	Contact Area	SASA	C-Score
361	SSSSSY*	-26.2	0.82	70	0	191	-45.45
188	SASSSQ*	-24.8	0.82	70	0	194	-44.05
360	SSSSSS*	-24.6	0.82	70	0	192	-43.85
362	SSSSYG*	-24.6	0.841	79	0	204	-45.07
187	ASASSS*	-24.4	0.802	72	0	194	-43.58
359	GSSSSS*	-24.4	0	0	0	187	0
310	GSYNDF	-24.2	0.833	61	0	177	-42.75
301	GSGSNF*	-24	0.894	35	0	176	-40.86
302	SGSNFG*	-24	0.792	78	0	196	-43.63
243	GYNGFG*	-23.8	0.833	67	0	200	-43.00
320	NQSSNF	-23.6	0.732	90	0	208	-43.53
363	SSSYGS*	-23.6	0.796	60	0	187	-41.54
285	GSYDSY*	-23.4	0.835	56	0	177	-41.48
343	GGGQYF	-23.4	0.848	64	0	199	-42.52
358	GGSSSS*	-23.3	0.701	58	0	182	-39.57
282	GGSGSY*	-23.2	0.701	58	0	185	-39.47
197	SGSGNF*	-23.2	0.736	78	0	194	-42.04

Supplementary Table 2: Sequence of the Δamy+ LCD, lacking all predicted steric zipper regions (marked in yellow).

**B-LCD**	190 200 210 220 230 240<br>MASAS SSQRGRSGSG NFGGGRGGGF GGNDNFGRGG NFSGRGGFGG SRGGGGYGGS<br>250 260 270 280 290<br>GDGYNGFGND GGYGGGGPGY SGGSRGYGSG GQGYGNQGSG YGGSGSYDSY<br>300 310. 320 330 340<br>NNGGGGGFGG GSGSNFGGGG SYNDFGNYNN QSSNFGPMKG GNFGGRSSGP<br>350 360 370<br>YGGGGQYFAK PRNQGGYGGS SSSSYGSGR RF
**B-LCD-Δamy+**	10 20 30 40 50<br>MRGRGGGRGG GFGGNDNFGR GGNFSGRGGF GGSRGGGGYG GSGDNDGGYG<br>60 70 80 90 100<br>GGGPGYSGGS RGYGSGGQGY GNQGSGYNNG GGGGFGGGGG NYNGPMKGGN<br>110 120<br>FGGRSSGPYG AKPRNQGGYG RRF
