# Supplementary material for: The interface of condensates of the hnRNPA1 low-complexity domain promotes formation of amyloid fibrils
Source: Nat Chem. 2023 Sep 25;15(10):1340–9. doi: 10.1038/s41557-023-01289-9 (PMC10533390; doi:10.1038/s41557-023-01289-9)
Supplement: Supplementary file 2 — Reporting Summary [file 41557_2023_1289_MOESM2_ESM.pdf]

Corresponding author(s): Paolo Arosio
Last updated by author(s): Jun 6, 2023

Reporting Summary

Nature Portfolio wishes to improve the reproducibility of the work that we publish. This form provides structure for consistency and transparency in reporting. For further information on Nature Portfolio policies, see our Editorial Policies and the Editorial Policy Checklist.

Statistics

For all statistical analyses, confirm that the following items are present in the figure legend, table legend, main text, or Methods section.

n/a	Confirmed	
☐	☒	The exact sample size (*n*) for each experimental group/condition, given as a discrete number and unit of measurement
☐	☒	A statement on whether measurements were taken from distinct samples or whether the same sample was measured repeatedly
☒	☐	The statistical test(s) used AND whether they are one- or two-sided<br>*Only common tests should be described solely by name; describe more complex techniques in the Methods section.*
☒	☐	A description of all covariates tested
☒	☐	A description of any assumptions or corrections, such as tests of normality and adjustment for multiple comparisons
☐	☒	A full description of the statistical parameters including central tendency (e.g. means) or other basic estimates (e.g. regression coefficient) AND variation (e.g. standard deviation) or associated estimates of uncertainty (e.g. confidence intervals)
☒	☐	For null hypothesis testing, the test statistic (e.g. *F*, *t*, *r*) with confidence intervals, effect sizes, degrees of freedom and *P* value noted<br>*Give P values as exact values whenever suitable.*
☒	☐	For Bayesian analysis, information on the choice of priors and Markov chain Monte Carlo settings
☒	☐	For hierarchical and complex designs, identification of the appropriate level for tests and full reporting of outcomes
☒	☐	Estimates of effect sizes (e.g. Cohen's *d*, Pearson's *r*), indicating how they were calculated

Our web collection on statistics for biologists contains articles on many of the points above.

Software and code

Policy information about availability of computer code

Data collection	Widefield fluorescence / brightfield microscopy: MicroManager software (version 2.0 gamma)<br>Re-scan confocal microscopy: NIS Elements 5.20 Advanced research software (Nikon)<br>Plate reader software for Thioflavin T profiles: Reader Control and MARS softwares (BMG Labtech)<br>Raman spectra acquisition: LabVIEW 2020 software, Labspec 6.5, Multiwell, EasyNav (National Instruments)<br>Confocal microscopy: Leica LAS X SP8 software (version 1.0)<br>TEM Morgagni: iTEM 5.2 and MorgagniUI Version 3
Data analysis	Droplet size distributions and numbers: in-house written program in Matlab (version R2020a)<br>Image analysis: Fiji/ImageJ (version 2.3.0)<br>Raman spectroscopy analysis: Python (version 3.9.5), Wavemetrics IgorPro 9 software (version 9.01)<br>AFM: Nanoscope 8.1 software (Bruker)<br>All data were plotted using Matlab (version R2020a)<br>Steric zipper prediction: ZipperDB, https://services.mbi.ucla.edu/zipperdb/<br>LARKS prediction: LARKSdb, https://srv.mbi.ucla.edu/LARKSdb/index.py

For manuscripts utilizing custom algorithms or software that are central to the research but not yet described in published literature, software must be made available to editors and reviewers. We strongly encourage code deposition in a community repository (e.g. GitHub). See the Nature Portfolio guidelines for submitting code & software for further information.

Data

Policy information about availability of data

All manuscripts must include a data availability statement. This statement should provide the following information, where applicable:

- Accession codes, unique identifiers, or web links for publicly available datasets
- A description of any restrictions on data availability
- For clinical datasets or third party data, please ensure that the statement adheres to our policy

All source data relevant for this manuscript have been uploaded on Figshare and are publicly available under the following links:
https://doi.org/10.6084/m9.figshare.23262005.v1
https://doi.org/10.6084/m9.figshare.23262008.v1

Raw micrographs are available from the corresponding author upon request.
Used databases: ZipperDB (https://services.mbi.ucla.edu/zipperdb/), LARKSdb (https://srv.mbi.ucla.edu/LARKSdb/index.py)

Human research participants

Policy information about studies involving human research participants and Sex and Gender in Research.

Reporting on sex and gender	n.a.
Population characteristics	n.a.
Recruitment	n.a.
Ethics oversight	n.a.

Note that full information on the approval of the study protocol must also be provided in the manuscript.

Field-specific reporting

Please select the one below that is the best fit for your research. If you are not sure, read the appropriate sections before making your selection.

☒ Life sciences ☐ Behavioural & social sciences ☐ Ecological, evolutionary & environmental sciences

For a reference copy of the document with all sections, see nature.com/documents/nr-reporting-summary-flat.pdf

Life sciences study design

All studies must disclose on these points even when the disclosure is negative.

Sample size	No sample size calculation was performed. To represent data distribution, all experiments were carried out at least three times and depending on feasibility with respect to material availability
Data exclusions	No data was excluded
Replication	All experiments were repeated at least three times. Reproducibility was ensured by performing all experiments with different, independently produced protein batches yielding similar results. Data analysis was performed in an automated, unbiased manner. All findings were replicated and successfully reproduced. All shown micrographs are representative images.
Randomization	Samples were not allocated in different groups as this is a biophysical study which reports quantitative data and does not rely on comparison of experimental groups
Blinding	Experiments were not blinded. This study reports absolute quantitative data rather than aiming at comparing experimental groups. Therefore blinding was not required.

Reporting for specific materials, systems and methods

We require information from authors about some types of materials, experimental systems and methods used in many studies. Here, indicate whether each material, system or method listed is relevant to your study. If you are not sure if a list item applies to your research, read the appropriate section before selecting a response.

Materials & experimental systems

n/a	Involved in the study
☒	☐ Antibodies
☒	☐ Eukaryotic cell lines
☒	☐ Palaeontology and archaeology
☒	☐ Animals and other organisms
☒	☐ Clinical data
☒	☐ Dual use research of concern

Methods

n/a	Involved in the study
☒	☐ ChIP-seq
☒	☐ Flow cytometry
☒	☐ MRI-based neuroimaging
